# Symbiotic Bacterial Diversity, Functional Profiling and Antibiotic Susceptibility of the Red Imported Fire Ant

**DOI:** 10.3390/microorganisms14040808

**Published:** 2026-04-01

**Authors:** Yukang Xin, Lei Chen, Munazza Ijaz, Rui Chen, Natasha Manzoor, Alhassan Alrafaie, Xiao Wang, Jinyan Luo, Bin Li, Linfei Shou

**Affiliations:** 1 State Key Laboratory of Rice Biology and Breeding, Ministry of Agriculture and Rural Affairs Key Laboratory of Molecular Biology of Crop Pathogens and Insect Pests, Zhejiang Key Laboratory of Biology and Ecological Regulation of Crop Pathogens and Insects, Zhejiang Engineering Research Center for Biological Control of Crop Pathogens and Insect Pests, Zhejiang Provincial Key Laboratory of Agricultural Microbiomics, Institute of Biotechnology, Zhejiang University, Hangzhou 310058, China; 22416077@zju.edu.cn (Y.X.); drmunazzaijaz@zju.edu.cn (M.I.); natashafahd@gmail.com (N.M.); libin0571@zju.edu.cn (B.L.); 2 Department of Plant Quarantine, Shanghai Extension and Service Center of Agriculture Technology, Shanghai 201103, China; chenlei200524@163.com; 3 Hangzhou Agricultural Technology Extension Center (Hangzhou Plant Protection and Quarantine Center), Hangzhou 310020, China; chenrui0307@126.com; 4 Department of Medical Laboratory, College of Applied Medical Sciences in Al-Kharj, Prince Sattam bin Abdulaziz University, Al-Kharj 11942, Saudi Arabia; a.alrafaie@psau.edu.sa; 5 Ningbo Jiangbei District Agricultural Technology Extension Service Station, Ningbo 315020, China; smile-67@163.com; 6 Station for the Plant Protection & Quarantine and Control of Agrochemicals of Zhejiang Province, Hangzhou 310004, China

**Keywords:** *Solenopsis invicta*, symbiotic bacteria, diversity, functional profiling, pest management

## Abstract

The red imported fire ant (RIFA), *Solenopsis invicta*, is a globally invasive pest that causes substantial ecological, agricultural, and public health challenges. Conventional control strategies primarily depend on chemical insecticides, which present environmental risks and limited long-term efficacy. In this study, we comprehensively investigated the bacterial microbiota of *S. invicta* and compared it with a sympatric non-target ant species (*Pheidole nodus*) to explore the ecological significance and biocontrol potential of symbiotic bacteria. High-throughput 16S rRNA sequencing revealed that the symbiotic bacterial community of *S. invicta* exhibited markedly higher richness and diversity. A total of 1651 amplified sequence variants (ASVs) were identified, of which 1089 ASVs are unique to the RIFAs, and 460 are unique to non-target ants. Linear discriminant analysis effect size (LEfSe) highlighted 33 biomarker taxa (score > 6.5), with strong enrichment of *Stenotrophomonas*, *Serratia*, *Pseudomonas*, *Luteibacter*, *Bradyrhizobium*, *Brucella*, *Smaragdicoccus*, *Gordonia*, and *Aeromonas*. Functional predictions and enzymatic assays in vitro demonstrated that dominant cultivable genera, particularly *Stenotrophomonas* (SI-7, SI-17), *Serratia* (SI-1, SI-3, SI-6, SI-18), and *Pseudomonas* (SI-2, SI-8, SI-9, SI-11, SI-19), exhibit substantial proteolytic and lipolytic activity, suggesting key roles in nutrient metabolism and host ecological adaptability. Antibiotic susceptibility profiling further revealed that florfenicol shows broad-spectrum inhibitory activity against these dominant symbionts. These findings indicate that disrupting dominant symbiotic bacteria may impair host physiology and thus serve as a targeted control strategy. Overall, the study elucidates the diversity, functional potential, and biocontrol applicability of the *S. invicta* microbiome, providing a foundation for developing sustainable, microbiome-based pest management approaches.

## 1. Introduction

The red imported fire ant (RIFA), *Solenopsis invicta*, is a highly invasive and hazardous ant species belonging to the order Hymenoptera, family Formicidae, subfamily Myrmicinae, and genus *Solenopsis* [1]. It is widely recognized for its substantial ecological, agricultural, and economic impacts [2,3,4]. Native to the Paraná River basin in South America, *S. invicta* has primarily spread via international trade and cargo transport, and it has now invaded in numerous regions worldwide, including China, the southern United States, and India [5]. Since its initial detection in Guangdong Province in 2004, the infested area of RIFA in China has continued to expand [6]. Now it has spread to 673 counties (cities, districts) across 23 provinces (autonomous regions), including Zhejiang, Fujian, Guangdong, Guangxi, Hunan, Jiangxi, Sichuan, Guizhou, Yunnan, Hainan, and Taiwan [6,7]. The venomous sting of RIFA contains alkaloids that induce intense pain, serving as an effective deterrent against predators and aiding in prey capture [4]. However, this potent defense mechanism poses a significant public health risk, particularly for vulnerable groups such as the elderly, infants, and individuals in poor health [8,9]. Most individuals experience a sharp, pinprick-like pain following a RIFA sting, while allergic reactions may include redness, itching, and pustule formation [10,11,12,13]. In severe cases, anaphylactic shock or even death may occur [14]. Although RIFA can occasionally act as a pollinating insect, its invasion predominantly poses substantial threats to ecosystems and biodiversity [6,15]. Both laboratory and field studies have demonstrated that the presence of RIFA leads to marked declines in the richness and abundance of reptiles at regional scales [16]. Furthermore, RIFA attacks economically important crops such as wheat (*Triticum aestivum*), corn (*Zea mays*), and soybeans (*Glycine max*), resulting in root damage, reduced yields, as well as significant economic losses [17,18,19]. As a forestry pest, RIFA damages roots, shoots, flowers, and seeds, and adversely affects soil fertility and microbial diversity [20,21].

In recent years, host-associated microbial communities have been recognized as key factors in insect adaptation and successful invasion [22]. As core member of these communities, symbiotic bacteria extensively colonize various insect tissues, including the thorax, abdomen, salivary glands, digestive tract, fat body, hemolymph, and reproductive organs, and are even present in the Malpighian tubules, nervous system, and muscular system [22,23,24,25,26]. They play significant roles in host physiological processes such as nutrient metabolism, immune defense, and reproductive development [27,28]. Symbiotic bacteria provide insects with essential nutrients, including vitamins, lipids, and proteins, which significantly influence their growth and development [29,30]. Dysbiosis or insufficient abundance of these microbial communities often leads to reduced activity, developmental delays, and even diminished reproductive capacity in insects [31,32]. In addition to their nutritional role, symbiotic bacteria are crucial for enhancing their host’s resilience to environmental stressors, including defense against natural enemies. For instance, they inhibit the infection of foreign pathogenic microorganisms through redox mechanisms [33], assist aphids in resisting parasitoid wasps [34], and secrete toxic substances to deter predation by wolf spiders [35]. Moreover, studies suggest that symbiotic bacteria may be integral components of the host’s reproductive regulation system [36]. A well-known example is *Wolbachia*, which can induce cytoplasmic incompatibility between sperm and egg cells in various insects, including *Tribolium castaneum*, planthoppers, and parasitoid wasps [37,38,39]. This phenomenon is highly complex and correlated with factors such as *Wolbachia* strain, bacterial density, and host developmental stage [40,41].

Despite ongoing efforts, controlling the RIFA population remains a challenging task, primarily relying on the repeated application of chemical insecticides [42]. However, chemical control presents substantial limitations. Contact insecticides can poison non-target animals, while bait formulations tend to persist in the environment and accumulate through the food chain with unpredictable consequences, and long-term use of bait toxicants may lead to fire ant reinfestation [43,44]. Therefore, there is an urgent need to explore safer, more environmentally friendly, and sustainable pest management approaches [45]. Research on insects and their symbiotic bacteria has provided a novel perspective for pest control strategies, such as the utilization of *Wolbachia* for managing *Aedes aegypti* and the development of microbial insecticides like *Bacillus thuringiensis* (Bt) [46,47,48,49,50].

The primary objective of this study was to comprehensively characterize the bacterial microbiota associated with *S. invicta* and compare it with a sympatric non-target ant species to elucidate microbial factors underpinning their ecological adaptability. The research aimed to identify key biomarker taxa, assess functional capacities of dominant cultivable symbionts, and evaluate their roles in nutrient metabolism. Additionally, the study sought to determine the antibiotic susceptibility of dominant symbiotic bacteria, which explore the feasibility of microbiome-disruption-based, environmentally sustainable strategies for managing invasive fire ant populations.

## 2. Materials and Methods

### 2.1. Field Collection of Ant Samples

All ant samples were collected from highly infested areas in Jinhua, Zhejiang province, China. Five fire ant nests were excavated using a stainless-steel shovel. RIFAs were collected from each nest and transferred into 250 mL Erlenmeyer flasks containing 100 mL of sterile phosphate-buffered saline (PBS, 1×, pH 7.4), resulting in five RIFA samples originating from different nests, which were subsequently designated as Si1, Si2, Si3, Si4, and Si5. Concurrently, we excavated five nests of non-target ant (*P. nodus*) near the RIFA nests by using the same protocol. Non-target ant samples from each nest were collected into separate 250 mL Erlenmeyer flasks containing 100 mL of sterile PBS and were sequentially named Pn1, Pn2, Pn3, Pn4, and Pn5. Nest architecture and habitat of *S. invicta* and *P. nodus* were surveyed and photographed during sample collection. The dimensions of excavated ant nests were measured using a tape measure (DL280513L, Deli, Ningbo, China). Additionally, twenty individuals of RIFA and non-target ant were separately captured using fine forceps and transferred to 50 mL sterile centrifuge tubes for subsequent morphological observation. And morphological observations and image acquisitions were performed using an inverted microscope (C-DSS230, Nikon, Tokyo, Japan) equipped with a halogen lamp (6 V, 30 W) and a digital sight DS-Fi2 digital camera (K19493, Nikon, Tokyo, Japan). All collection equipment, including the stainless-steel shovels, Erlenmeyer flasks, and fine forceps, underwent surface decontamination via ultraviolet (UV) irradiation for 60–90 min prior to field use. Immediately following collection, all samples were aseptically transferred into sterile plastic bags. To preserve biological integrity and prevent shifts in microbial community structure, samples were maintained on ice packs within insulated containers during transport and processed immediately upon arrival at the laboratory.

### 2.2. Identification and Storage of Ant Samples

All collected ant samples were transferred to new 10 mL sterile centrifuge tubes, followed by the addition of 8 mL of sterile PBS. Following 3 consecutive washes with PBS for 1 min each, the ant samples were further disinfected by immersing them into 5 mL of sterile 70% (*v*/*v*) ethanol and then incubating for 50 s. The ethanol solution was rapidly aspirated, and the samples were rinsed thoroughly 3 times with sterile deionized water to eliminate residual ethanol, which could inhibit subsequent microbial analyses. The surface-sterilized ant samples were partitioned into aliquots within new sterile centrifuge tubes and cryovials. One set of aliquots were sent to the Shanghai Lingen Biotechnology Co., Ltd. (Shanghai, China) for species identification, using the universal primers LCO1490 (Forward primer: 5′-GGTCAACAAATCATAAAGATATTGG-3′) and HCO2198 (Reverse primer: 5′-TAAACTTCAGGGTGACCAAAAAATCA-3′) [51]. Another set of aliquots was designated for immediate downstream analyses, including microbiome profiling and bacterial isolation procedures. The remaining aliquots were flash-frozen in liquid nitrogen for 5 min to ensure rapid vitrification and then transferred to a −80 °C ultra-low temperature freezer for long-term archival storage. Aseptic technique was maintained throughout all processing steps.

### 2.3. Evaluation of Difference in Symbiotic Bacterial Community Structure and Composition

To assess the differences in symbiotic bacterial community structure and composition between RIFAs and non-target ants, the following methodology was employed: Approximately 500–600 ants were taken from each pre-processed ant sample, and symbiotic bacterial community DNA extraction was performed using the E.Z.N.A.^®^ Stool DNA Kit (Omega Bio-tek, Norcross, GA, USA). The V4–V5 region of 16S rRNA gene was PCR-amplified with 515F (5′-GTGYCAGCMGCCGCGGTAA-3′) and 907R (5′-CCGTCAATTCMTTTRAGTTT-3′) primers. The PCR reactions were carried out in 20 μL volume with the 4 μL of 5× FastPfu Buffer, 2 μL of dNTPs (2.5 mM), 0.8 μL of Forward primer (5 μM), 0.8 μL of Reverse primer (5 μM), 0.4 μL of FastPfu Polymerase, 10 ng of template DNA, and 12 μL ddH_2_O. The PCR was carried out using the following steps: 95 °C 5 min; 95 °C 30 s, 55 °C 30 s, 72 °C 30 s, 25 cycles; 72 °C 5 min. The PCR amplification products were sent to the Shanghai Lingen Biotechnology Co., Ltd. for high-throughput sequencing on the Illumina PE300. Following the sequencing process, raw reads underwent quality control using FASTP (v0.20.0) [52]. Paired-end reads were then merged into longer single sequences using FLASH (v1.2.11) [53]. The raw reads was further processed using the DADA2 algorithm, including quality filtering, denoising, merging, and removal of chimeric sequences, and the generation of amplicon sequence variants (ASVs) and feature tables [54]. The phylogenetic affiliation of each ASV was analyzed by UCLUST algorithm against the SILVA (SSU138.2) 16S rRNA database using confidence threshold of 80% [55,56].

### 2.4. Isolation and Identification of Bacterial Symbionts from RIFAs

Surface-sterilized RIFA samples were crushed in 2 mL of sterile PBS. Subsequently, the homogenate was subjected to a brief sedimentation period of 3 min at 25 °C to allow coarse particulate matter to settle. The supernatant obtained after sedimentation was designated as the undiluted extract (10^0^). Serial ten-fold dilutions (10^−1^ to 10^−5^) of this extract were prepared in sterile PBS. A 100 μL suspension with each dilution was smeared onto surface of Tryptic Soy Agar (TSA) plates. The TSA medium composition per liter was: pancreatic digest of casein (15 g), peptic digest of soybean meal (5 g), sodium chloride (5 g), agar (15 g), dissolved in distilled water (final pH 7.2 ± 0.2), and sterilized by autoclaving at 121 °C for 15 min. Inoculated plates were incubated aerobically at 28 °C for 24 h. Following incubation, discrete bacterial colonies exhibiting varying morphologies were selected. Pure bacterial strains were obtained by performing 3 successive streak-transfer isolations onto fresh TSA plates, with incubation at 28 °C for 24 h between each transfer. These strains were then used for downstream analyses, specifically colony morphology observation and 16S rRNA gene sequencing for taxonomic identification, extracellular enzyme activity detection as well as antibiotic susceptibility testing. All purified bacterial strains were preserved by storing them in a 20% glycerin solution at a temperature of −80 °C in an ultra-low refrigerator for future use.

To identify the culturable symbiotic bacteria associated with RIFA, representative isolates were characterized by 16S rRNA gene sequencing and analysis [57]. In brief, the 16S rRNA genes of the isolated strains were amplified using the universal primers 27F (5′-AGAGTTTGATCCTGGCTCAG-3′) and 1492R (5′-GGTTACCTTGTTACGACTT-3′). The PCR reaction mixture (50 μL) included ddH_2_O (18 μL), 2× Hieff^®^ Ultra-Rapid II HotStart PCR Master Mix* (25 μL), 10 μM forward primer (2 μL), 10 μM reverse primer (2 μL), and 0.5–0.8 OD_600_ bacterial suspension (3 μL). The PCR was carried out using the following steps: 95 °C 3 min; 95 °C 15 s, 56 °C 20 s, 72 °C 30 s, 35 cycles; 72 °C 5 min. PCR products were visualized on 1.0% agarose gels, then purified and submitted to Tsingke Biotechnology Co., Ltd., Hangzhou, China for sequencing in both directions. All the obtained 16S rDNA sequences were aligned using ClustalX (version 2.1) software to remove redundant sequences with high similarity. Following alignment, the sequence was submitted to nucleoside BLAST (https://blast.ncbi.nlm.nih.gov/Blast.cgi, accessed on 21 March 2026) of NCBI for homology alignment, with a threshold similarity of >97% to determine the taxonomic affiliation of the strains [58,59]. Subsequently, a phylogenetic tree was reconstructed using the Maximum Likelihood method in MEGA 11 software based on closely related sequences of the isolated strains. The reliability of the phylogenetic tree was evaluated through bootstrap analysis with 1000 replicates [60].

### 2.5. Assaying Extracellular Protease and Lipase Activities of Isolated Bacterial Strains

After 24 h of activation on TSA plates at 28 °C, a single colony was selected from each bacterial strain and incubated in Tryptic Soy Broth (TSB, identical in composition to the TSA but without agar) at 28 °C and 200 rpm for 18 h in a shaking incubator. The resulting bacterial suspension was adjusted to a density of 10^7^ CFU per mL. NA medium was heated to 50–55 °C and supplemented with either skim milk (RM00014, ABclonal, Wuhan, China) at 1.5% (*w*/*v*) or tributyrin (T0364, Tokyo Chemical Industry, Tokyo, Japan) at 1% (*v*/*v*) to prepare plates for the detection of extracellular protease and lipase, respectively. The NA medium consisted of tryptone (10 g), glucose (2.5 g), beef extract (3 g), NaCl (5 g), agar (15 g), and distilled water (1000 mL; pH 7.0), and sterilized by autoclaving at 121 °C for 15 min. Aliquots (2.5 μL) of the bacterial suspensions were spotted onto extracellular enzyme detection plate, and each strain was assayed in triplicate. Incubate the test bacteria at 28 °C for 36 h and observe the formation of a transparent halo around the colony.

### 2.6. Antibiotic Susceptibility Assessment of Symbiotic Bacteria

The selection of antibiotics was guided by the objective of disrupting the symbiotic bacterial community within RIFAs as a potential strategy for pest management. In the study, ten antibiotics representing eight major classes were selected, including aminoglycosides (kanamycin, gentamicin, streptomycin), β-lactams (penicillin), quinolones (fleroxacin), macrolides (roxithromycin), tetracyclines (tetracycline), amphenicols (florfenicol), carbapenems (imipenem), and rifamycins (rifampin). Collectively, these antibiotics cover multiple mechanisms of action, including the inhibition of protein synthesis (aminoglycosides, tetracyclines, macrolides, and amphenicols) [61,62,63,64], cell wall synthesis (β-lactams and carbapenems) [65,66], nucleic acid synthesis (fluoroquinolones) [67], and RNA transcription (rifamycins) [68]. The aim was to establish a systematic basis for evaluating the susceptibility of culturable bacteria associated with RIFAs and to provide a foundation for subsequent in vivo assays assessing the impact of antibiotic exposure on symbiotic bacterial community and host fitness. The Kirby–Bauer disk diffusion method was employed for antimicrobial susceptibility testing [69]. The Mueller–Hinton agar (MHA) medium (WP8470, G-CLONE, Beijing, China) used in the experiments consisted of soluble starch (1.5 g), beef extract (5 g), acid hydrolysate of casein (1.5 g), agar (12.5 g), and ddH_2_O (1000 mL). The pH value was adjusted to 7.3, and the medium was sterilized by autoclaving at 121 °C for 15 min. Using a sterile inoculating loop, 2–3 isolated colonies from purified bacterial cultures grown on TSA plates were transferred into 5 mL of sterile physiological saline (0.9% NaCl). The bacterial suspension was mixed thoroughly, and its turbidity was adjusted to 0.5 McFarland standard using a turbidity standard tube (YA0180, Solarbio^®^, Beijing, China). A sterile cotton swab was dipped into the adjusted suspension and used to evenly inoculate the entire surface of the MHA plate. This process was repeated 2–3 times, rotating the plate approximately 90° each time to ensure uniform distribution. Finally, the rim of the agar was swabbed to ensure complete coverage. After the surface moisture was fully absorbed by the agar, sterile forceps were used to apply antibiotic disks (6 mm in diameter, 0.7 mm in thickness, BKMAMLAB, Changsha, China) onto the agar surface. Each disk was gently pressed to ensure firm contact with the medium. Each bacterial isolate was tested in triplicate. The inoculated plates were left at room temperature for 20–30 min to allow pre-diffusion of the antibiotics. Subsequently, the plates were incubated at 28 °C for 24 h. Following incubation, the diameters of the inhibition zones were measured using the cross method and recorded in millimeters (mm). Due to the current absence of a unified interpretive standard for inhibition zones specifically applicable to environmental isolates, this study referred to the principles of the disk diffusion method as outlined in the Performance Standards for Antimicrobial Susceptibility Testing, 36th Edition [70]. Based on these principles and combined with the distribution characteristics of inhibition zone diameters, the results were classified as follows: an inhibition zone diameter > 6 mm (greater than the diameter of the disk) was considered susceptible, whereas an inhibition zone diameter ≤ 6 mm, or the absence of an inhibition zone (recorded as 0 mm), was considered resistant. The Cluster heatmap of antibiotic sensitivity of 15 isolated strains were plotted using R (version 4.5.1).

### 2.7. Statistical Analysis

Rarefaction analysis based on Mothur v.1.21.1 was performed to reveal alpha diversity indices, including the Chao1, ACE, Pielou’s Evenness, and Shannon indices [71]. Differences in these indices between groups were assessed using Student’s *t*-test. Beta diversity was evaluated based on Bray–Curtis distances, which were used to perform principal co-ordinates analysis (PCoA) and hierarchical clustering [72,73]. The hierarchical clustering tree was constructed by using unweighted pair group method with arithmetic mean (UPGMA) [74]. Permutational multivariate analysis of variance (PERMANOVA) was performed using R vegan package to assess the statistically significant effects of bacterial microbiota among different ant samples. Visualization of relative abundances (RAs) of ASVs as well as dominant bacterial and fungal taxa at the phylum and genus levels presented in bar plots, heatmaps, and bubble plots were conducted using R (version 4.5.1). Differential analysis of gut bacterial communities between red imported fire ant samples and other ant samples at the phylum and genus levels were performed using the Mann–Whitney U test. Linear discriminant analysis effect size (LEfSe) was employed to identify differentially abundant taxa from phylum to species level, and the magnitude of the effect of each taxon’s abundance was evaluated based on linear discriminant analysis (LDA) scores [75]. Phylogenetic Investigation of Communities by Reconstruction of Unobserved States (PICRUSt2) program based on the Kyoto Encyclopedia of Genes and Genomes (KEGG) database was used to predict the functional alteration of bacterial microbiota in different ant samples [76].

## 3. Results

### 3.1. Morphological and Molecular Identification of Ant Samples

**Figure 1:**
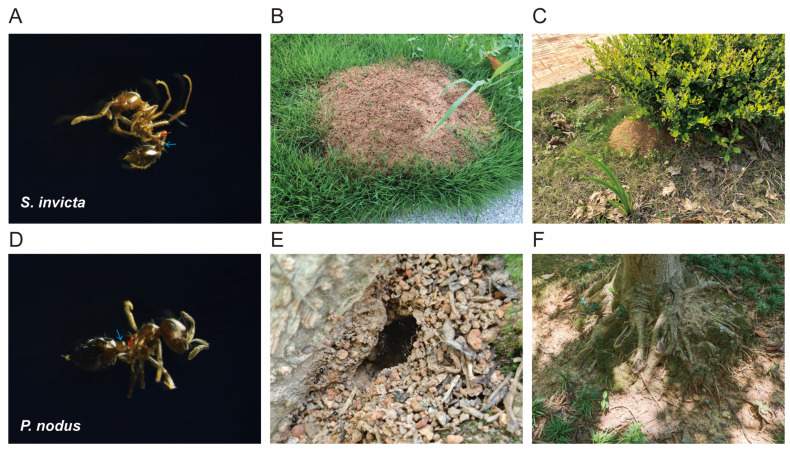
Morphology and habitat environment between *Solenopsis invicta* and *Pheidole nodus.* (**A**,**D**) Morphology of a single ant observed by inverted microscope; (**B**,**E**) Nest structure; (**C**,**F**) Habitat; Petiole and postpetiole have been marked with red and blue arrows, respectively.

To clarify the species identity of the ant samples in this study, both molecular and morphological identification were performed on two groups of specimens (Figure 1). Under microscopic examination, the petiole and postpetiole of the ants in group 1 are similar in size, a morphological characteristic matching that of *S. invicta* (Figure 1A). In contrast, the postpetiole of the ants in group 2 is distinctly swollen and significantly larger than the petiole, a feature consistent with that of *P. nodus* (Figure 1D). BLAST analysis of the mitochondrial cytochrome coxidase subunit 1 (CO1) gene sequences on NCBI revealed that the CO1 sequence of Group 1 ants (GenBank: PX474348) showed 100% similarity to that of *S. invicta*, while Group 2 ants (GenBank: PX474349) shared 99.40% similarity with the CO1 gene sequence of *P. nodus* (Table S1). Additionally, comparisons of nest structures and habitats showed that Group 1 ants built large nests with distinct dome-shaped mounds (approximately 10–45 cm in height) above the ground. The internal structure of these nests was honeycomb-like, with no clearly visible entrance holes (Figure 1B). These nests were predominantly distributed in open, sun-exposed areas such as lawns, farmlands, and parks (Figure 1C). On the other hand, Group 2 ants typically established nests in shallow soil, near tree roots, or within cracks in pavements-environments that were moist and covered (Figure 1F). Their nests were relatively small and simply structured, with clearly observable entrance (Figure 1E). Collectively, these results confirmed that the ant samples in Group 1 were *S. invicta*, while those in Group 2 were *P. nodus*.

### 3.2. Differences in Symbiotic Bacterial Community Between S. invicta and Non-Target Ants

**Figure 2:**
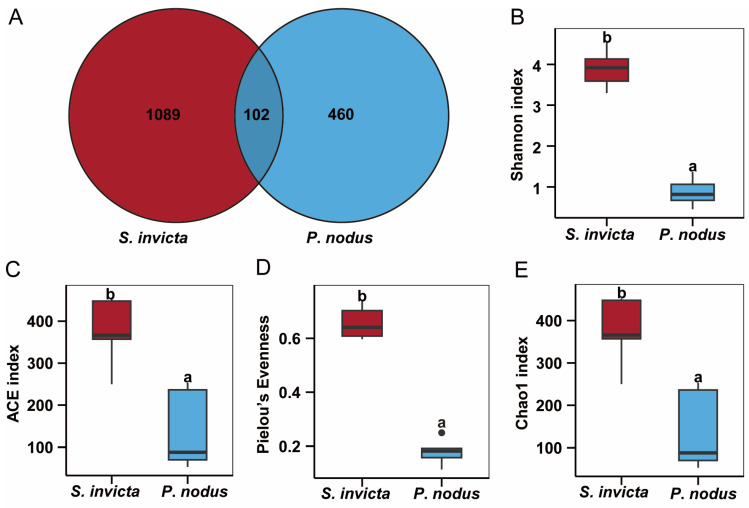
ASVs distribution and alpha diversity of symbiotic bacterial community between the *Solenopsis invicta* and *Pheidole nodus*. (**A**) Illustrates the differences in the distribution of ASVs; (**B**) Shannon; (**C**) ACE; (**D**) Pielou’s Evenness; (**E**) Chao1; Statistically significant differences among groups (*p* < 0.05, Student’s *t*-test) were indicated with different superscript lowercase letters.

Based on the high-throughput amplicon sequencing, a total of 1651 bacterial ASVs were identified from 2 groups of ant samples (*n* = 5), and 102 bacterial ASVs were distributed in both groups (Figure 2). Differences were observed in the number of bacterial ASVs between RIFAs (*S. invicta*) and non-target ants (*P. nodus*) (Figure 2A). Indeed, the average number of unique bacterial ASVs in *P. nodus* was 460 (proportion 27.86%), while in *S. invicta* it was 1089 (proportion 65.96%). This indicates that the relative abundance of symbiotic bacteria in *S. invicta* is higher than that in *P. nodus*. Furthermore, the α-diversity (including Shannon, ACE, Pielou’s Evenness, and Chao1 analysis) was performed to evaluate the species richness and diversity of symbiotic bacteria in 10 ant samples (Figure 2B–E). Indeed, the average bacterial Chao1, ACE, Shannon, and Pielou’s Evenness indices of *S. invicta* were 374.61, 374.69, 3.90, and 0.66, respectively, whereas the corresponding indices for *P. nodus* were 140.01, 140.23, 0.87, and 0.18. Additionally, there was a significant difference in the α-diversity of symbiotic bacterial community between *S. invicta* and *P. nodus* (*p* < 0.05), which indicated that the symbiotic bacterial richness and diversity in RIFAs was significantly higher than that in non-target ants.

**Figure 3:**
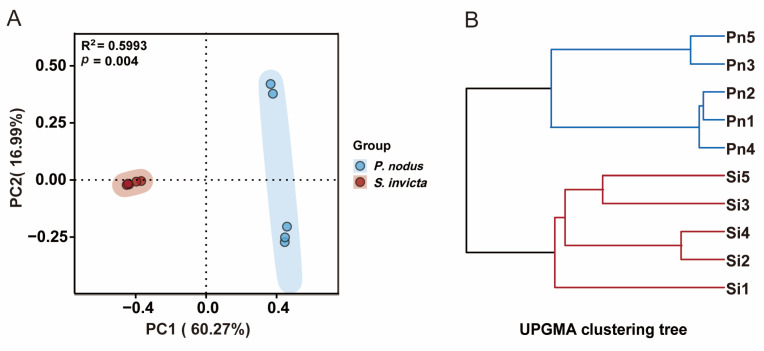
Beta diversity of symbiotic bacterial community between the *Solenopsis invicta* (Si) and *Pheidole nodus* (Pn). (**A**) PCoA; (**B**) Hierarchical cluster analysis; Ellipses are included in the plot, indicating the 0.95 confidence limit; The red and blue branches cluster Si samples and Pn samples, respectively.

To assess the similarity and dissimilarity of symbiotic bacterial communities in ant samples, the Bray–Curtis metric was employed to evaluate β-diversity visualized by hierarchical cluster analysis as well as PCoA. Based on the symbiotic bacterial community structures, all samples were divided into two distinct groups, suggesting that there were obvious differences in the bacterial community compositions of *S. invicta* and *P. nodus* (Figure 3B). Consistent with the above findings, the bacterial ASV abundance in *S. invicta* and *P. nodus* samples also exhibited an evident two-group clustering pattern (Figure 3A). Among them, the first principal component contributed 60.27% to the sample differences, and the second principal component contributed 16.99% (PERMANOVA: R^2^ = 0.5993; *p* = 0.004). Overall, there were significant differences in symbiotic bacterial community compositions between RIFAs and non-target ants.

**Figure 4:**
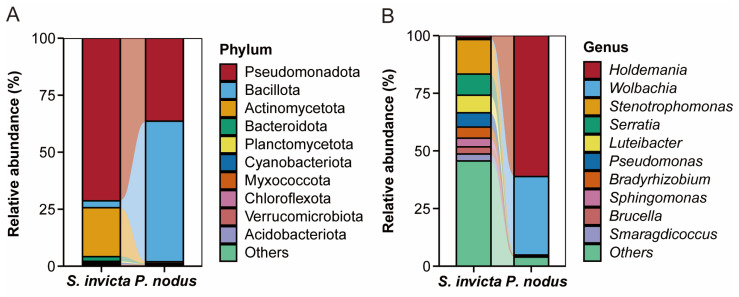
Relative abundances of symbiotic bacterial community between the *Solenopsis invicta* and *Pheidole nodus*. (**A**) Phylum level; (**B**) Genus level.

**Figure 5:**
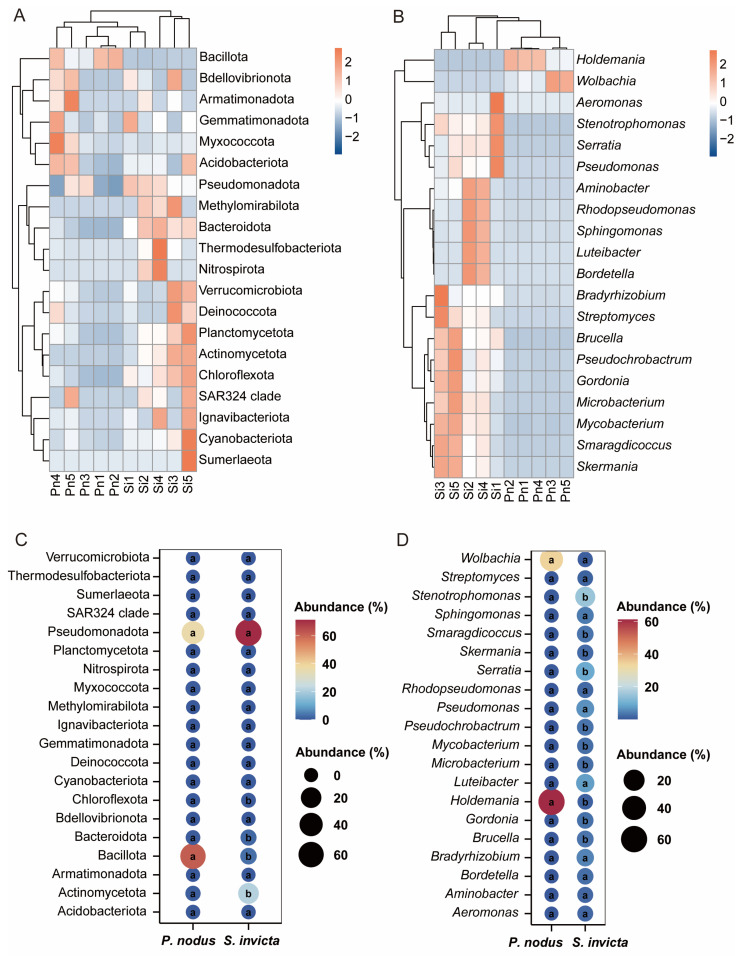
Differences in symbiotic bacterial community composition and relative abundance between *Solenopsis invicta* (Si) and *Pheidole nodus* (Pn). (**A**,**B**) Differences in bacterial community composition at the phylum and genus levels; (**C**,**D**) Differences in bacterial community relative abundance at the phylum and genus levels; Statistically significant differences among groups (*p* < 0.05, Student’s *t*-test) indicated with different superscript lowercase letters.

To provide a clearer representation of the disparities in symbiotic bacterial communities between RIFAs and non-target ants, a histogram of relative abundance at the top 10 phylum and genus levels was carried out (Figure 4). The results indicated that the microbial community structure of *S. invicta* varied from that of *P. nodus*. The dominant phyla of bacterial community in *P. nodus* were Bacillota (61.68%) and Pseudomonadota (36.37%). In *S. invicta*, Pseudomonadota (71.32%), Actinomycetota (21.44%), Bacillota (3.04%), and Bacteroidota (2.06%) were the dominant phyla of bacterial community (Figure 4A). Comparison at the genus level revealed distinct differences in bacterial community abundance between ant species (Figure 4B). The microbiota in *S. invicta* was characterized by diverse dominant genera, including *Stenotrophomonas* (14.99%), *Serratia* (9.18%), *Luteibacter* (7.62%), *Pseudomonas* (6.17%), *Bradyrhizobium* (4.83%), *Sphingomonas* (3.78%), *Brucella* (3.08%), and *Smaragdicoccus* (3.02%). In contrast, *P. nodus* exhibited a significantly less diverse community structure, dominated almost exclusively by *Holdemania* (61.15%) and *Wolbachia* (34.00%). Furthermore, heat maps and bubble plots visually demonstrate the differences in symbiotic bacterial communities between *S. invicta* and *P. nodus* (Figure 5). Notably, while the bacterial community structures (including composition and relative abundance) at the phylum level exhibited similarities between RIFA and non-target ant samples (Figure 5A,C), significant divergence was observed at the genus level (Figure 5B,D).

**Figure 6:**
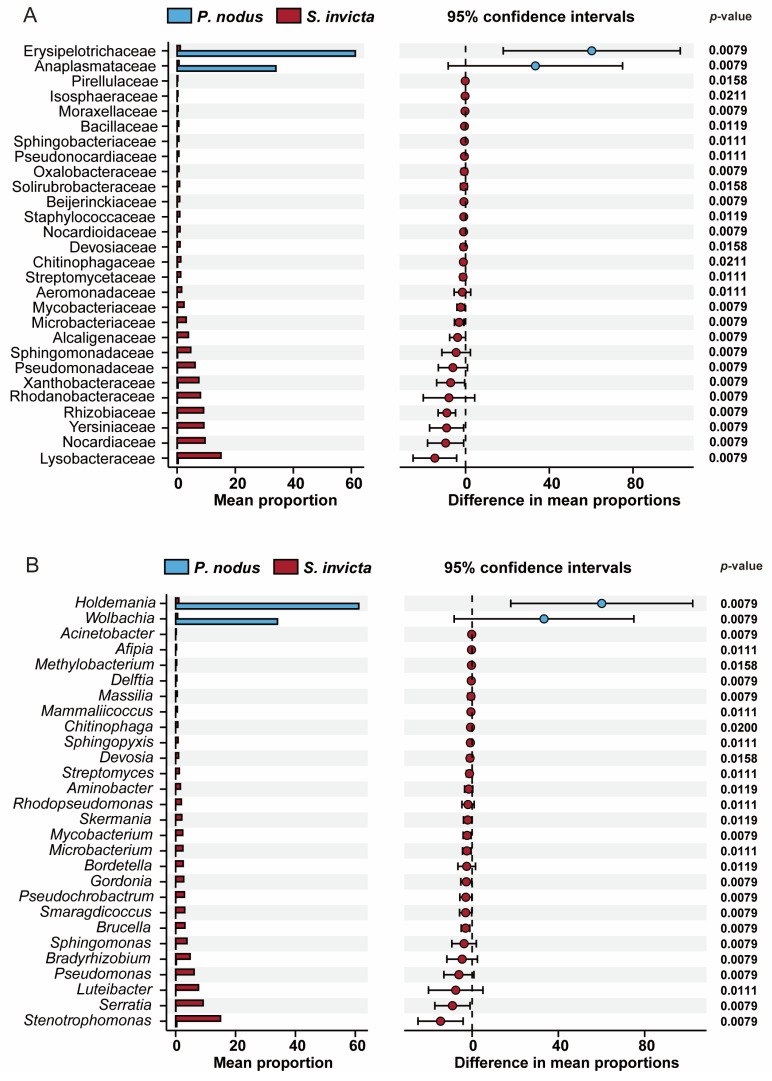
Significant difference analysis of symbiotic bacterial communities in ant samples (*p* < 0.05, Mann–Whitney U test). (**A**) Family level; (**B**) Genus level.

To identify symbiotic bacteria exhibiting significant differences between RIFAs and non-target ants, differential species analysis was performed. This analysis revealed distinct bacterial taxa at both the family and genus levels (*p* < 0.05; Mann–Whitney U test) (Figure 6). At the family level, the symbiotic bacterial communities in RIFAs were significantly enriched in Lysobacteraceae, Nocardiaceae, Yersiniaceae, Rhizobiaceae, Rhodanobacteraceae, Xanthobacteraceae, Pseudomonadaceae, and Sphingomonadaceae compared to those in non-target ants (Figure 6A). Conversely, the symbiotic bacterial communities in non-target ants demonstrated significantly greater abundance of Erysipelotrichaceae and Anaplasmataceae (Figure 6A). Genus-level analysis showed that *Holdemania* and *Wolbachia* were significantly more abundant in non-target ants (Figure 6B). In contrast, RIFA communities exhibited significantly higher abundances of *Stenotrophomonas*, *Serratia*, *Luteibacter*, *Pseudomonas*, *Bradyrhizobium*, *Sphingomonas*, *Brucella*, and *Smaragdicoccus* (Figure 6B).

**Figure 7:**
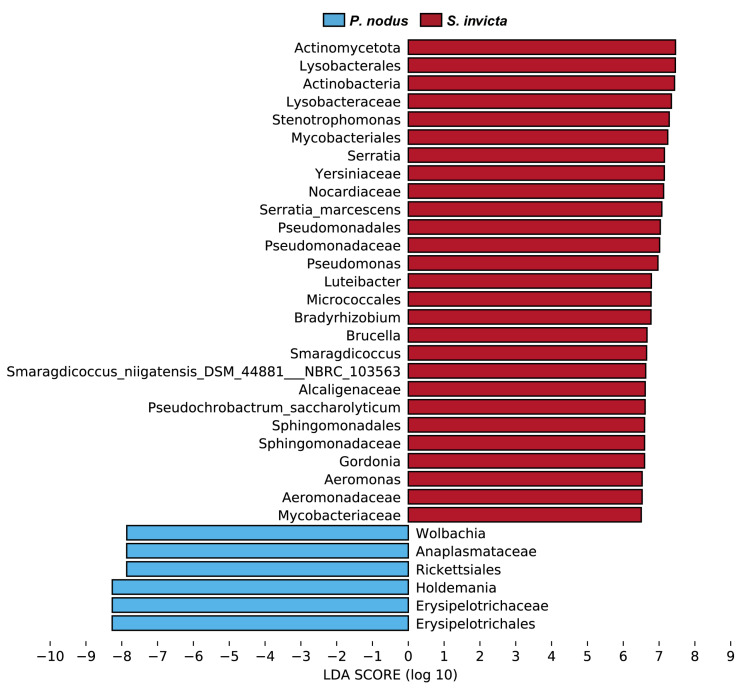
The effect size of Liner discriminant analysis (LDA) on the bacterial taxa. Only bacterial taxa with LDA values > 6.5 are displayed (*p* < 0.05, non-parametric factorial Kruskal–Wallis and sum-rank test).

LEfSe was employed to identify differentially abundant bacterial taxa between RIFAs and non-target ants, spanning taxonomic levels from phylum to genus. The LDA score was used to estimate the magnitude of the effect of each differentially abundant species. The analysis revealed 33 bacterial biomarkers (LDA > 6.5) significantly associated with either *S. invicta* or *P. nodus* groups (Figure 7). Comparatively richer in distinctive bacterial taxa, the RIFAs exhibited biomarker genera primarily including *Stenotrophomonas*, *Serratia*, *Pseudomonas*, *Luteibacter*, *Bradyrhizobium*, *Brucella*, *Smaragdicoccus*, *Gordonia*, and *Aeromonas*. In contrast, the distinctive microbiota of non-target ants predominantly consisted of the genera *Holdemania* (family Erysipelotrichaceae, order Erysipelotrichales) and *Wolbachia* (family Anaplasmataceae, order Rickettsiales).

**Figure 8:**
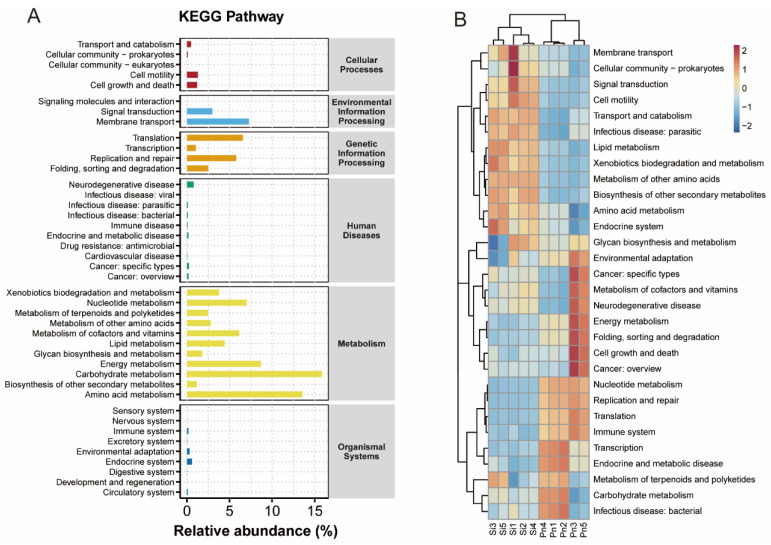
Relative abundances of KEGG pathways at level 2 based on 16S rRNA gene sequences from *Solenopsis invicta* (Si) and *Pheidole nodus* (Pn). (**A**) Relative abundances of KEGG pathways at level 2 from all ant samples; (**B**) Differences in functional profiles and abundance patterns in the bacterial microbiota between *S. invicta* and *P. nodus*.

To investigate the potential functional roles of symbiotic bacteria in RIFAs (*S. invicta*) and non-target ants (*P. nodus*), PICRUSt was employed to predict the relative abundances of KEGG pathways at level 2 based on 16S rRNA gene sequences from all ant samples (Figure 8). Analysis of KEGG pathway abundances revealed five predominant functional categories: carbohydrate metabolism, amino acid metabolism, energy metabolism, membrane transport, and nucleotide metabolism (Figure 8A). Among the top 20 most abundant metabolic pathways, other significant functions included translation, replication and repair, metabolism of cofactors and vitamins, lipid metabolism, xenobiotics biodegradation and metabolism, and signal transduction (Figure 8A). Heatmap analysis revealed distinct functional profiles and abundance patterns in the bacterial microbiota of RIFAs compared to non-target ants (Figure 8B). The symbiotic bacterial community within *S. invicta* was predominantly associated with metabolic pathways related to membrane transport, cellular community in prokaryotes, signal transduction, cell motility, transport and catabolism, infectious parasitic diseases, lipid metabolism, xenobiotics biodegradation and metabolism of other amino acids, biosynthesis of other secondary metabolites, and the endocrine system (Figure 8B). In contrast, the microbiota of *P. nodus* showed significant enrichment in pathways involved in nucleotide metabolism, replication and repair, translation, immune system functions, transcription, as well as endocrine and metabolic diseases (Figure 8B).

### 3.3. Identification and Phylogenetics of Bacterial Symbionts in S. invicta

**Figure 9:**
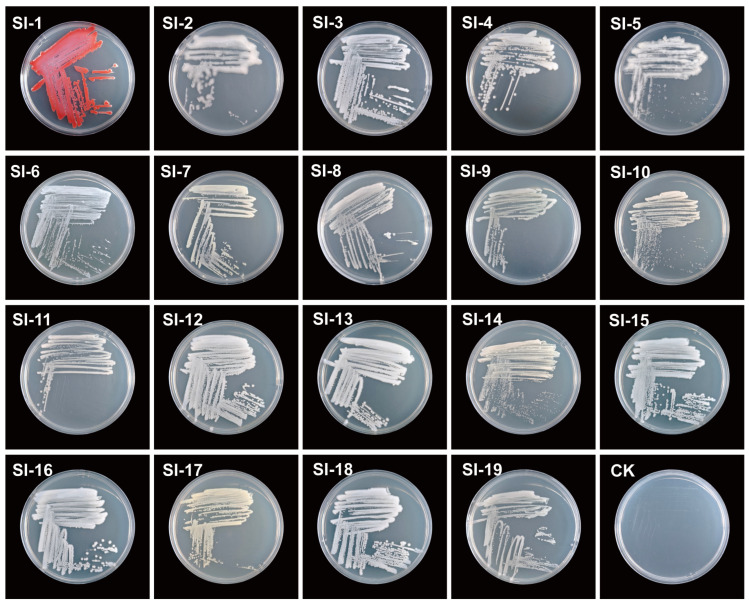
The 19 strains of culturable symbiotic bacteria obtained from the internal tissues of *Solenopsis invicta*.

**Figure 10:**
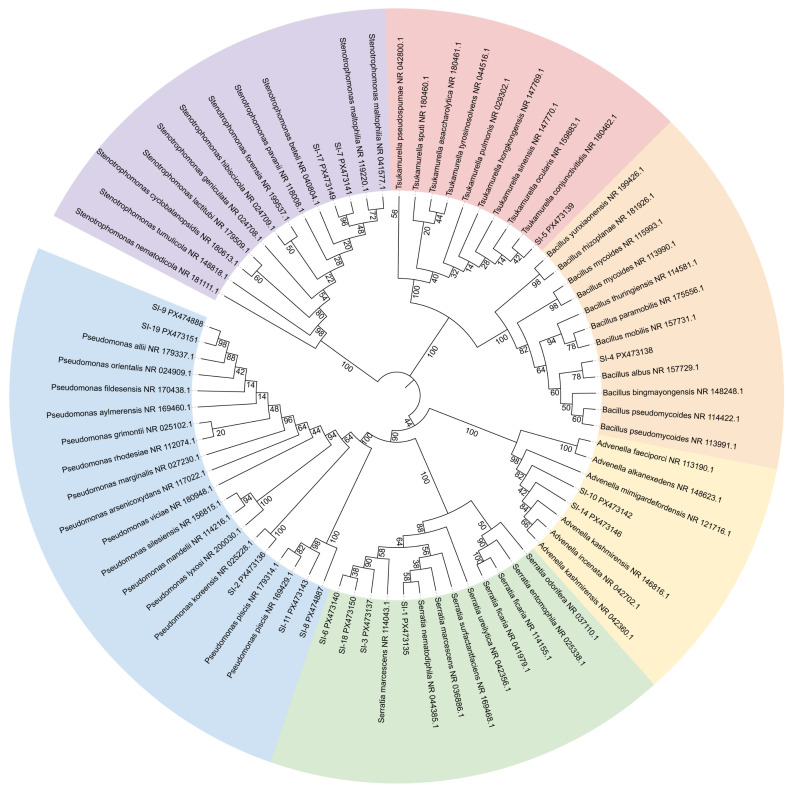
Phylogenetic tree of 16S rRNA in cultivable symbiotic bacteria of the internal tissues of *Solenopsis invicta*.

To better clarify the composition and structural characteristics of the symbiotic bacterial community within RIFAs, we performed observation of colony morphology, 16S rRNA sequencing and phylogenetic tree construction of the culturable bacteria isolated from the host. A total of 19 symbiotic bacterial strains were isolated in this study (Figure 9; Table 1). Colonies of SI-1 were red, round, convex, and viscid, with a smooth surface, as well as an entire margin. SI-2, SI-4, SI-8, SI-9, SI-11, and SI-19 colonies were round, dirty white, smooth, and viscid, with an entire margin. SI-3, SI-6, SI-12, SI-13, SI-15, SI-16 and SI-18 colonies were round, white, convex, and viscid, with a smooth surface, as well as an entire margin. Colonies of SI-5 were dirty white, irregular, and powdery, with a rough surface, as well as a serrate margin. SI-7, SI-10, and SI-14 colonies were round, pale yellow, smooth, and viscid, with an entire margin. Colonies of SI-17 were round, smooth, as well as viscid, with a yellow color and an entire margin. The molecular sequencing results indicate that 15 distinct sequences were obtained. After removing redundant sequences through Clustal multiple sequence alignment (Table 1). Similarity comparison analysis revealed that strains SI-7 and SI-17 showed 99.66% sequence similarity and were identified as *Stenotrophomonas maltophilia*. And 99.73% sequence similarity was shown between *Serratia marcescens* and strains SI-3 as well as SI-6. Similarly, strains SI-1 and SI-18 exhibited 99.86 and 99.80% sequence similarity, respectively, and were also identified as *Serratia marcescens*. Strains SI-8 and SI-11 shared 99.93% sequence similarity and were identified as *Pseudomonas piscis*. Strains SI-9 and SI-19 showed 99.50% and 99.79% sequence similarity, respectively, and were identified as *Pseudomonas allii*. Strains SI-10 and SI-14 showed 99.64% and 99.57% sequence similarity, respectively, and were identified as *Advenella kashmirensis*. Furthermore, strains SI-2, SI-5, and SI-4 were identified as *Pseudomonas koreensis*, *Tsukamurella ocularis*, and *Bacillus albus*, respectively, with sequence similarities of 99.79%, 99.93%, and 99.93%, as indicated (Table 1). Phylogenetic analysis indicated that these 15 different strains belonged to the phyla Pseudomonadota, Bacillota, and Actinomycetota, with Pseudomonadota being the dominant phylum, accounting for 86.67%, while Bacillota and Actinomycetota represented smaller proportions, each at 6.67% (Figure 10). The 13 strains within the phylum Pseudomonadota were classified into 4 genera: 5 strains of *Pseudomonas* (SI-2, SI-8, SI-9, SI-11, SI-19), 4 strains of *Serratia* (SI-1, SI-6, SI-3, SI-18), 2 strains of *Advenella* (SI-10 and SI-14), and 2 strains of *Stenotrophomonas* (SI-7 and SI-17). Then, SI-5 was identified as *Tsukamurella* genus within the phylum Actinomycetota, as well as SI-4 was identified as *Bacillus* genus within the phylum Bacillota.

### 3.4. Detection of Extracellular Protease and Lipase Activities in Symbiotic Bacteria

**Figure 11:**
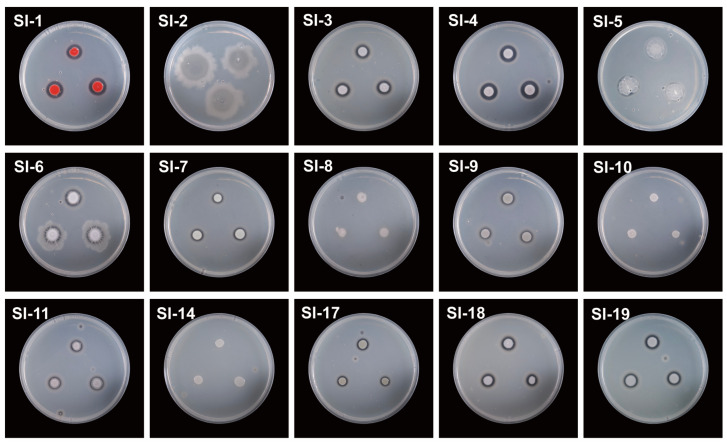
Extracellular protease production assays of 15 strains on NA media containing 1.5% skim milk (showing the production of clear zone after 36 h of incubation).

**Figure 12:**
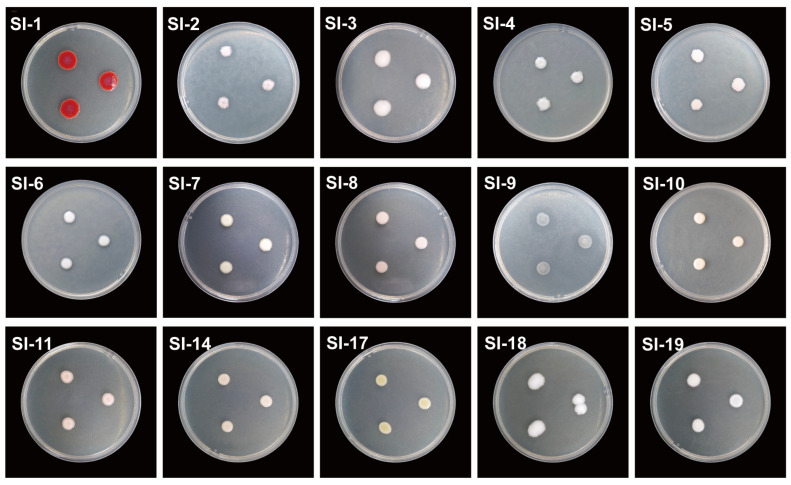
Extracellular lipase production assays of 15 strains on NA media containing 1% tributyrin (showing the production of clear zone after 36 h of incubation).

Additionally, in this study extracellular protease and lipase activity assays were evaluated to explore the potential role of symbiotic bacterial communities in host RIFAs (Figure 11 and Figure 12; Table 1). The results indicated that there were differences in protease production among the 15 strains when grown on nutrient agar (NA) supplemented with 1% skim milk. Specifically, no protease production was observed for strains SI-2, SI-5, SI-8, SI-10, and SI-14, as evidenced by the absence of clear zones around the colonies (Figure 11). In contrast, distinct clear zones were observed around strains SI-1, SI-3, SI-4, SI-6, SI-7, SI-9, SI-11, SI-17, SI-18, and SI-19, indicating that these strains can produce extracellular proteases during growth (Figure 11). Additionally, in the lipase activity assay, clear zones were observed around strains SI-2, SI-5, SI-6, and SI-9, demonstrating their ability to produce lipases (Figure 12). However, no evident clear zones were detected around the remaining 12 strains, suggesting that these bacteria do not produce lipases during growth (Figure 12).

### 3.5. Antibiotic Susceptibility Assessment of Symbiotic Bacteria from S. invicta

**Figure 13:**
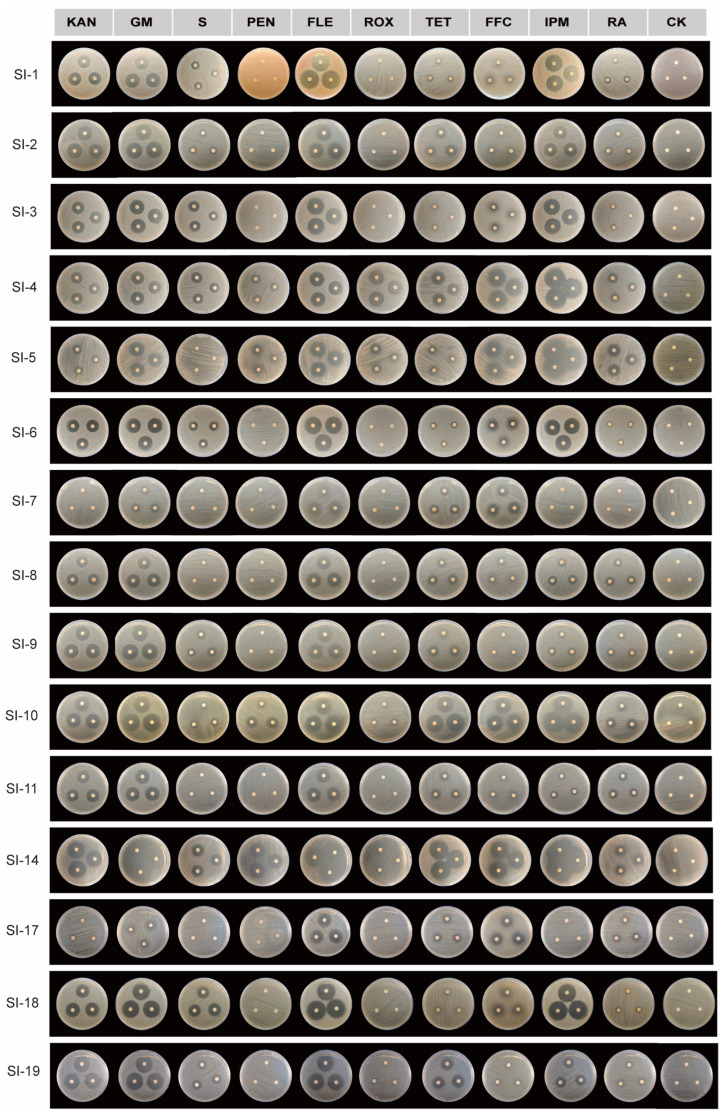
Antibiotic sensitivity assays of 15 strains on MHA media (showing the production of clear zone after 24 h of incubation). KAN: kanamycin, GM: gentamicin; S: streptomycin; PEN: penicillin; FLE: fleroxacin; ROX: roxithromycin; TET: tetracycline; FFC: florfenicol; IPM: imipenem; RA: rifampin.

To develop more effective control strategies, we evaluated the susceptibility of culturable symbiotic bacteria isolated from red imported fire ants to 10 antibiotics including kanamycin (KAN), gentamicin (GM), streptomycin (S), penicillin (PEN), fleroxacin (FLE), roxithromycin (ROX), tetracycline (TET), florfenicol (FFC), imipenem (IPM), and rifampin (RA). The results revealed varying levels of susceptibility among the 15 bacterial strains to different antibiotics (Table 2; Figure 13). Strains SI-7 (6 mm) and SI-17 (6 mm) were identified as non-susceptible to kanamycin. In contrast, KAN susceptibility was observed in strains SI-1 (21.92 mm), SI-2 (23.64 mm), SI-3 (20.72 mm), SI-4 (19.46 mm), SI-5 (14.14 mm), SI-6 (20.71 mm), SI-8 (19.57 mm), SI-9 (24.32 mm), SI-10 (24.24 mm), SI-11 (20.64 mm), SI-14 (28.09 mm), SI-18 (23.48 mm), and SI-19 (26.96 mm). All 15 strains exhibited susceptibility to both GM and FLE. Notably, strain SI-7 produced a diffuse inhibition zone around the fleroxacin disk. Resistance to S was detected in strains SI-5, SI-7, SI-8, SI-11, and SI-17. The remaining strains were susceptible to S, with inhibition zone diameters as follows: SI-1 (12.50 mm), SI-2 (12.23 mm), SI-3 (17.59 mm), SI-4 (17.86 mm), SI-6 (14.28 mm), SI-9 (14.30 mm), SI-10 (12.21 mm), SI-14 (18.28 mm), SI-18 (20.22 mm), and SI-19 (15.91 mm). PEN susceptibility was observed only in strains SI-4 (13.52 mm), SI-5 (11.26 mm), SI-10 (18.67 mm), and SI-14 (25.01 mm). The other 11 strains were non-susceptible to PEN. A similar pattern was observed for ROX, to which strains SI-4 (22.98 mm), SI-5 (17.49 mm), SI-10 (7.88 mm), and SI-14 (10.46 mm) were susceptible, while the remaining strains showed resistance. TET susceptibility testing indicated that only strain SI-18 was non-susceptible. The following strains were susceptible to tetracycline: SI-1 (8.33 mm), SI-2 (16.83 mm), SI-3 (6.72 mm), SI-4 (21.17 mm), SI-5 (10.68 mm), SI-6 (8.45 mm), SI-7 (16.68 mm), SI-8 (17.06 mm), SI-9 (13.76 mm), SI-10 (29.03 mm), SI-11 (10.99 mm), SI-14 (32.28 mm), SI-17 (13.65 mm), and SI-19 (18.62 mm). For FFC, susceptibility was detected in strains SI-1 (12.25 mm), SI-3 (11.95 mm), SI-4 (28.06 mm), SI-5 (29.10 mm), SI-6 (12.61 mm), SI-7 (17.99 mm), SI-10 (24.69 mm), SI-11 (9.13 mm), SI-14 (28.34 mm), SI-15 (19.45 mm), SI-18 (10.19 mm), and SI-19 (11.59 mm). In contrast, strains SI-2, SI-8, and SI-9 were non-susceptible. IPM susceptibility was observed in 13 strains, with inhibition zones measuring 28.67 mm (SI-1), 22.28 mm (SI-2), 26.80 mm (SI-3), 35.99 mm (SI-4), 37.83 mm (SI-5), 26.61 mm (SI-6), 15.13 mm (SI-8), 13.66 mm (SI-9), 36.03 mm (SI-10), 11.99 mm (SI-11), 44.36 mm (SI-14), 31.83 mm (SI-18), and 18.02 mm (SI-19). Strains SI-7 and SI-17 were non-susceptible to IPM. Additionally, RA resistance was observed in strain SI-7. The following strains were susceptible to RA: SI-1 (10.67 mm), SI-2 (11.95 mm), SI-3 (8.68 mm), SI-4 (16.72 mm), SI-5 (20.30 mm), SI-6 (8.91 mm), SI-8 (15.12 mm), SI-9 (11.91 mm), SI-10 (17.23 mm), SI-11 (15.22 mm), SI-14 (16.56 mm), SI-17 (13.34 mm), SI-18 (7.00 mm), and SI-19 (12.96 mm).

**Figure 14:**
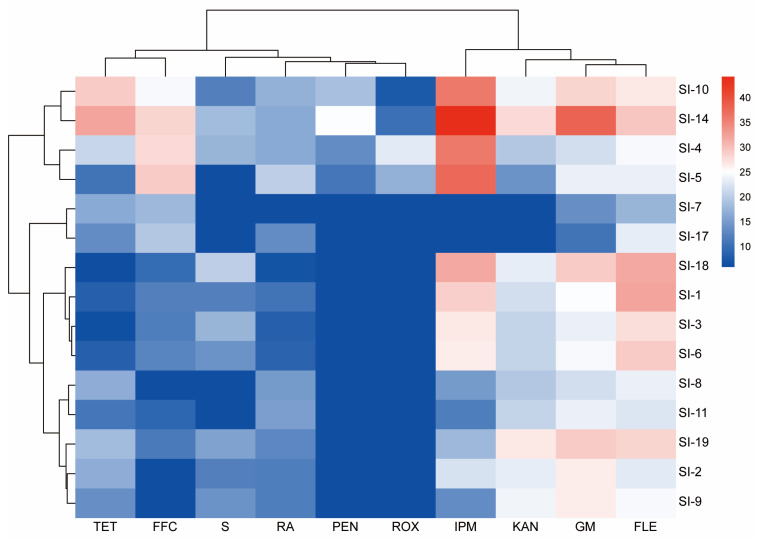
The Cluster heatmap of antibiotic sensitivity of 15 symbiotic bacterial strains.

Furthermore, clustering analysis of the susceptibility profiles of culturable symbiotic bacteria from RIFAs to 10 antibiotics revealed that strains belonging to the same genus, as identified by 16S sequencing, generally exhibited similar antibiotic susceptibility patterns (Figure 14). Specifically, strains SI-8, SI-11, SI-9, SI-2, and SI-19 of the genus *Pseudomonas* displayed highly comparable susceptibility profiles and clustered within a single small branch. Similarly, strains SI-18, SI-1, SI-3, and SI-6 of the genus *Serratia* were grouped into another distinct subclade. In contrast, the antibiotic susceptibility profiles of *Stenotrophomonas* strains SI-7 and SI-17 differed remarkably from those of the above groups and formed a separate cluster. Strains of *Tsukamurella* (SI-4), *Bacillus* (SI-5), and *Advenella* (SI-10, SI-14) were grouped together into a larger branch. Notably, their antibiotic susceptibility patterns were substantially different from those of the other 11 strains, particularly in terms of their responses to FFC and IPM. Additionally, clustering of the antibiotic effects on the culturable bacterial isolates indicated that FLE, GM, IPM and KAN exhibited the broadest antibacterial spectra among the 10 antibiotics tested.

## 4. Discussion

### 4.1. Microbiome as a Key Driver of Ecological Adaptability in the Invasion of RIFAs

In this study, we employed high-throughput sequencing to characterize the symbiotic bacterial communities associated with the RIFAs and sympatric non-target ants (*P. nodus*). Our results demonstrated that the symbiotic bacterial community within *S. invicta* exhibits significantly greater richness and diversity than that of *P. nodus*, a pattern potentially linked to its remarkable invasive success and adaptability. Animal microbiomes are known to mediate critical host phenotypic traits, including energy metabolism, reproduction, and immunity [77]. For invasive species, the acquisition and maintenance of a highly plastic or diverse microbial community can facilitate rapid adaptation to novel environmental conditions [78,79,80]. Supporting this notion, the gut microbiome is associated with the successful colonization of spiders in the Hawaiian Islands [81]. It is important to note that the relationship between the microbiome and animal invasion is complex and bidirectional, as the microbial composition of the invader can also be shaped by the biotic and abiotic characteristics of the local ecosystem [82]. Nonetheless, our findings contribute to deeper understanding the role of the microbiome in invasion ecology. Subsequent research should build upon this work by incorporating more refined measures of host fitness and employing advanced analytical approaches to elucidate the underlying mechanisms.

Principal Coordinates Analysis and histogram analyses further revealed substantial dissimilarities in symbiotic bacterial composition between *S. invicta* and the non-target ants at both the phylum and genus levels. Notably, the relative abundance of Actinobacteria was significantly higher in *S. invicta*, which may be closely related to the antibiotic-producing capabilities of this phylum [83,84]. Intriguingly, *S. invicta* appears to exhibit a specific preference for Actinobacteria. Newly mated queens utilize olfactory cues to locate Actinobacteria-rich soil for nest foundation [85], and workers show a preference for such soil during nest expansion [86]. Furthermore, fire ant colonization significantly increases the abundance of Actinobacteria in nest soils [87,88]. Collectively, these pieces of evidence suggest a complex mutualistic relationship between *S. invicta* and Actinobacteria. The ants’ active olfactory-driven selection of Actinobacteria-rich soil provides the colony with natural antibiotic protection, potentially suppressing pathogenic microbes and thereby enhancing colony health and survival. In return, ant settlement activities, such as the deposition of waste, glandular secretions, and nest structure modification that alter the microhabitat, significantly promote the proliferation and enrichment of actinobacteria within the nest soil.

At the genus level, the symbiotic bacterial community in the RIFAs was characterized by a diversity of dominant genera, including *Stenotrophomonas* (14.99%), *Serratia* (9.18%), *Luteibacter* (7.62%), *Pseudomonas* (6.17%), *Bradyrhizobium* (4.83%), *Sphingomonas* (3.78%), *Brucella* (3.08%), and *Smaragdicoccus* (3.02%). In contrast, the bacterial assemblage in the non-target ant species was dominated almost exclusively by *Holdemania* (61.15%) and *Wolbachia* (34.00%). Species composition and LEfSe analysis further identified these bacterial genera as distinctive biomarker taxa for *S. invicta* and the non-target ants (*P. nodus*), respectively. *Stenotrophomonas* is often regarded as an opportunistic pathogen in animals and humans, causing infections in immunocompromised hosts [89,90]. However, it also plays a positive role in plastic-eating *Tenebrio molitor* by promoting the absorption and utilization of polyvinyl chloride [91]. Certain strains may also participate in the degradation of insecticides, thereby aiding host resistance to chemical pesticides [92]. The genus *Serratia* is well-recognized as an entomopathogen and has been utilized as a bio-insecticide [93,94,95]. Recent evidence suggests that these bacteria may also act as opportunistic gut symbionts, assisting the host in reducing the toxicity of organophosphate pesticides [96,97]. *Luteibacter* is commonly found in soil and the plant rhizosphere, but its presence in insects remains poorly studied and is generally hypothesized to result from environmental acquisition [98,99]. It has been detected in the gut of *Aegosoma sinicum*, though its functional role remains unclear [100]. *Pseudomonas* is an exceptionally diverse genus, encompassing well-known entomopathogens such as *P. aeruginosa*, *P. fluorescens*, and *P. syringae* [101,102,103], as well as beneficial endosymbionts including *P. melophthora*, *P. adelgestsugas*, and pederin-producing *Pseudomonas* strains in insects [104,105,106]. *Bradyrhizobium* is best known for its nitrogen-fixing symbiosis with leguminous plants; however, its role within insect hosts remains largely unexplored [107,108]. It is speculated that ants may acquire this bacterium from soil, where it could transiently or persistently colonize the gut and potentially serve as a nitrogen source for the host [109,110].

The genus *Sphingomonas* has been identified in various insects, including scale insects, the yellow-legged hornet (*Vespa velutina*), and the coffee berry borer (*Hypothenemus hampei*) [111,112,113], though its functional roles remain poorly defined. Recent evidence indicates that gut-associated *Sphingomonas* can enhance the metabolism of imidacloprid in the cotton aphid (*Aphis gossypii*), confirming a detoxification capacity for this genus in insects [114]. *Brucella*, a genus of significant veterinary importance as the causative agent of brucellosis is typically considered a transient or weak symbiont in insects and may induce disease under certain conditions [115,116]. *Smaragdicoccus*, a relatively understudied genus initially isolated from soil, has been shown to colonize maize seedling roots in a manner influenced by the western corn rootworm [117,118]. Furthermore, no prior studies have reported the presence of *Smaragdicoccus* within insects; it is thus hypothesized to be a transient bacterium in *S. invicta*, potentially acquired from the rhizosphere soil.

*Holdemania* is widely distributed in the human gut, where it is thought to co-regulate nutrient metabolism and immune function alongside other gut microbes, though its precise role remains unclear [119,120]. Based on available data, it is speculated that *Holdemania* may contribute to nutrient metabolism in the non-target ant species, acting as a constituent of the gut microbiota [121]. In contrast, *Wolbachia*, a well-studied intracellular symbiont widespread among insects, is primarily known for manipulating host reproduction [122,123]. Recent studies demonstrate that *Wolbachia* can significantly enhance host resistance to viral pathogens and is currently employed in biocontrol strategies against mosquito-borne diseases [124,125,126,127]. Furthermore, the distribution of dominant microbial taxa within *S. invicta* was systematically compared across distinct geographic regions (Table 3). The study revealed significant differences in microbial composition colonizing different ant body parts, including the gut, venom gland, and hemolymph [128,129,130,131]. And only bacteria belonging to the genus *Bacillus* were detected in the hemolymph [129]. It is noteworthy that bacteria from the genera *Pseudomonas*, *Bacillus*, *Serratia*, *Stenotrophomonas* and *Bradyrhizobium* were widely associated with *S. invicta*, which aligns with our findings [128,129,130,131,132,133,134]. Compared to the non-target ant *P. nodus*, species within the genus *Solenopsis* generally harbored a richer microbial diversity [128,130,131,132,134]. Certainly, expanding the scope of analysis to include a broader range of RIFA and non-target ant species across the country would greatly enhance the universality and value of our research.

However, the structure of symbiotic bacterial community of *S. invicta* is often influenced by the collected samples, collection locations, and collection methods. In addition, more biological experiments need be carried out to verify the functions of symbiotic bacteria. In summary, *S. invicta* has a highly diverse and abundant symbiotic bacterial community, which are closely associated with its robust social immunity, broad omnivorous diet, and the improved adaptive demands of invasion. The fire ant microbiome serves as an extended genome, substantially augmenting its resilience and adaptability to environmental variation. This microbial advantage constitutes a key factor enabling *S. invicta* to outcompete native ant species and establish itself as a dominant invasive organism.

### 4.2. Culturable Symbiotic Bacteria in RIFAs Exhibit Substantial Metabolic Capabilities

KEGG functional prediction based on 16S rRNA gene sequencing of ant samples indicated that dominant symbiotic bacteria in the RIFAs are associated with nutrient metabolism pathways. These include lipid metabolism, amino acid metabolism, xenobiotic biodegradation and metabolism, biosynthesis of secondary metabolites, glycan biosynthesis and metabolism, and metabolic pathways related to the endocrine system. During the isolation and cultivation of symbiotic bacteria from *S. invicta*, we observed that all strains of *Stenotrophomonas* (SI-7, SI-17), *Serratia* (SI-1, SI-3, SI-6, SI-18), and *Pseudomonas* (SI-2, SI-9, SI-11, SI-19) genera except strain SI-8 exhibited robust proteolytic or lipolytic capabilities. This functional congruence underscores the potential critical role of culturable symbiotic bacteria in the host’s nutritional physiology. Throughout long-term coevolution, insects and their microbiota have established interdependent symbiotic relationships [26]. Insects provide a stable habitat and essential nutrients for symbiotic bacteria, while these bacteria participate in various metabolic processes of the host [135]. For instance, in the brown planthopper (*Nilaparvata lugens*) and termites, certain symbiotic bacteria can recycle nitrogenous waste into reusable amino acids, thereby enhancing the host’s nutritional status [136,137,138]. In aphids, the symbiotic bacteria *Buchnera* produces several essential amino acids that facilitate host survival and development [27]. Furthermore, some gut symbiotic bacteria synthesize diverse enzymes, such as cellulases and hemolysins, which aid in the digestion and absorption of nutrients [139,140]. Similarly, dominant symbiotic bacteria in the RIFAs have been confirmed to produce extracellular proteases and lipases. These enzymes are likely to assist the host in breaking down indigestible dietary proteins and complex proteins from other insect carcasses, converting them into amino acids that provide essential nutrients for larval growth and queen reproduction. They may also participate in the degradation, synthesis, or transformation of lipids, thereby supporting the ecological adaptability and broad omnivory of *S. invicta* across various niches [18,141,142].

Furthermore, we isolated a strain of *Tsukamurella* (SI-5) capable of degrading both proteins and lipids. *Tsukamurella*, a genus of actinomycetes known to cause ocular and respiratory infections in humans, includes some strains that are beneficial to plants, such as those that solubilize phosphorus to promote peanut seedling growth [143,144,145]. Research on *Tsukamurella* in insects remains limited, though a recent study identified members of this genus in social wasps with broad potential for antibiotic biosynthesis [146]. The specific role of *Tsukamurella* in *S. invicta* remains unclear. It is hypothesized that this bacterium may act as an opportunistic commensal. Under normal conditions, it may aid in the digestion of proteins and lipids, but it could potentially cause infection and host mortality if immune defenses are compromised. From a broader perspective, the influence of symbiotic bacteria on insects is complex and multifaceted, and not always beneficial. While symbiotic bacteria can contribute to host nutrition, the host must also allocate substantial energy and resources to provide a protected microenvironment for these microbes. Symbionts may protect the host through competition, antibiotic production, and immune activation; however, they can also turn into opportunistic pathogens under conditions of host immunosuppression [23]. The outcomes of insect-bacteria interactions depend on a dynamic equilibrium shaped by complex adaptations among the bacteria, the host, and the environment. Our study systematically validates the predicted metabolic functions of cultivable symbiotic bacteria in *S. invicta*, providing experimental evidence that clarify their symbiotic roles.

Our study detected multiple symbiotic bacterial genera, including *Stenotrophomonas*, *Serratia*, *Pseudomonas*, *Luteibacter*, *Bradyrhizobium*, *Sphingomonas*, *Brucella*, and *Smaragdicoccus*, within the microbiome of *S. invicta* through high-throughput sequencing. However, pure cultures of all targeted bacterial genera were not obtained during the separation and cultivation process. This discrepancy can be attributed to several common factors in microbial ecology, primarily revolving around cultivation limitations, microbial physiological states, and ecological interdependencies [147]. A key limiting factor is the mismatch between standard laboratory media and the specific nutritional requirements of many fastidious bacteria. For instance, *Bradyrhizobium* species are renowned for their extremely slow growth and often require specific symbiotic cues for optimal proliferation [148,149]. Bacteria within the *Luteibacter* genus typically thrive in oligotrophic conditions, which are not met by nutrient-rich TSA [150]. *Sphingomonas* species can be poor competitors and are easily overgrown by faster-growing microbes on non-selective media [151]. Similarly, *Smaragdicoccus* bacteria grow slowly and are suitable for survival on TSA medium with low nutrients [117]. Notably, *Brucella* species have stringent and complex nutritional demands, generally requiring supplementation with animal serum or blood to provide essential growth factor [152,153,154]. Beyond nutritional constraints, 16S rRNA gene sequencing may detect DNA signals of dormant or dead bacteria, which are difficult to isolate and cultivate. Furthermore, the existence of certain bacteria may depend on the unique host microenvironment or their interactions with other microorganisms, making it difficult for them to grow independently without this environment. But it is worth mentioning that we successfully isolated strains belonging to the first three genera *Stenotrophomonas*, *Serratia*, and *Pseudomonas*, which validates the reliability of the 16S rRNA gene sequencing data for these cultivable taxa.

Nevertheless, certain limitations should be acknowledged. Cultivable bacteria represent only a fraction of the total microbial community in insects, and the potential role of non-cultivable and difficult to cultivate bacteria is not yet clear. In future research, personalized culture media that simulate host environments (such as adding ant body extracts), extending culture time, or using co-culture techniques to isolate these bacteria will be considered. In addition, multilocus sequence analysis (MLSA) in each available genus should be applied in the identification process of cultivable symbiotic bacteria. For example, *groEL* gene is regarded as an effective target for species-level identification of *Tsukamurella* [155]. Housekeeping gene *gyrA* is used for the identification of bacteria belonging to the genus *Bacillus* [156]. For the genus *Pseudomonata*, the combined sequencing analysis of *rpoD*, *gyrB*, and *rpoB* genes can help us identify new strains at the species level [157]. In addition, *gyrB* gene is also used to differentiate and identify the species of *Stenotrophomonas* [158]. About the genus *Serratia*, *gyrB*, *rpoB*, *infB* and *atpD* gene sequences has been successfully applied for the classification of *Serratia* species [159,160]. And species identification of *Advenella* isolates is mainly based on 16S gene sequencing results, sometimes supplemented by morphological, phenotypic, or phylogenetic feature data [161]. Of course, whole-genome sequencing should be considered in future studies to provide more accurate bacterial classification for bacterial species that MLSA cannot differentiate. In addition, the metabolic functions verified in this study under in vitro conditions may not fully reflect the complex metabolic pathways operating within the host environment. Future studies should include in vivo functional validation, metabolomic profiling, and genetic manipulation approaches to further elucidate the mechanistic roles of symbiotic bacteria in *S. invicta*.

### 4.3. Novel Strategies and Future Perspectives for Sustainable Control of RIFAs

In this study, in vitro antibiotic susceptibility testing was conducted to preliminarily assess the potential of targeting symbiotic bacteria for controlling red imported fire ant populations. The results revealed that the dominant symbiotic bacterial genera *Stenotrophomonas* (SI-7, SI-17), *Serratia* (SI-1, SI-3, SI-6, SI-18), and *Pseudomonas* (SI-2, SI-8, SI-9, SI-11, SI-19) in *S. invicta*, exhibited varied susceptibility to 10 different antibiotics. Notably, the amphenicol antibiotic FLE demonstrated potent inhibitory effects against nearly all tested strains. FLE is a broad-spectrum veterinary antibiotic whose mechanism of action involves reversible binding to the bacterial 50S ribosomal subunit, thereby effectively inhibiting protein synthesis [162,163]. Previous experiments have indicated that these dominant symbiotic bacteria play crucial roles in insect nutrition and metabolism. Consequently, disruption of their stable symbiotic bacterial communities via FLE may induce physiological dysfunction in *S. invicta*, offering a promising approach for population control.

The RIFA is a globally significant invasive pest that poses serious threats to ecosystems and human health [164]. Numerous attempts have been made to develop environmentally friendly and sustainable control strategies against this pest [45]. For instance, botanical extracts such as ethanol extract of *Sophora flavescens* roots, essential oils from the bark and leaves of *Cinnamomum loureirii*, and essential oils and active compounds from Lamiaceae plants have been evaluated for RIFA control [165,166,167]. These plant-derived extracts generally exhibit lower environmental toxicity and reduced impacts on human health. Recently, insect cadavers infected with entomopathogenic nematodes and the fungal agent *Beauveria bassiana* strain Bb04 have also shown potential for suppressing RIFA populations [168,169].

Furthermore, the establishment of RNAi-based methods for RIFA management has provided a theoretical foundation for developing RNAi-derived nucleic acid pesticides [170]. In agreement with Wolbachia-mediated reproductive manipulation in rice planthoppers [171], our study targets dominant symbiotic bacteria within RIFA offers a novel approach for population control. However, translating these experimental findings into practical applications requires careful consideration. First, further in vivo validation is needed to assess the delivery efficiency, stability, and actual inhibitory efficacy of antibiotics in live ant colonies. Second, several critical risks associated with the application of antibiotics such as FLE must also be considered. For instance, the development of resistant bacteria may lead to unpredictable physiological consequences, potentially complicating population control efforts. Additionally, broad-spectrum antibiotics pose certain risks to non-target organisms, including beneficial soil microbiota and insects, and may result in contamination of soil and water systems, thereby disrupting ecosystem functions. Therefore, future research should explore the integration of FLE with chemical insecticide baits, or consider more targeted delivery systems and more specific intervention measures, such as species-specific baits and bacteriophages. Such integrated approaches may facilitate the development of more sustainable and resistance-resilient management strategies for RIFA.

## 5. Conclusions

This study provides a comprehensive characterization of the symbiotic bacterial microbiota associated with the RIFA *S. invicta*, revealing fundamental differences from the sympatric non-target ant *P. nodus*. The markedly higher bacterial richness and diversity observed in *S. invicta*, along with the identification of 33 biomarker taxa, highlight the unique microbial signatures that likely contribute to its exceptional ecological adaptability and invasive success. Dominant symbiotic genera, including *Stenotrophomonas*, *Serratia*, and *Pseudomonas*, demonstrated strong proteolytic and lipolytic capacities, underscoring their involvement in nutrient acquisition, metabolic regulation, and possibly immune modulation within the host. These functional attributes suggest that the microbiome acts as an extended physiological system supporting the ant’s survival across heterogeneous environments. The antibiotic susceptibility assays further indicated that florfenicol effectively targets the major cultivable symbionts of *S. invicta*, offering a promising direction for developing microbiome-disruption-based control strategies. However, the translation of these findings into field applications requires extensive in vivo validation, optimized delivery systems, and evaluation of potential resistance development. Collectively, this study provides new insights into the ecological roles of symbiotic bacteria in *S. invicta* and establishes a scientific basis for designing sustainable, symbiont-targeted approaches for managing this invasive pest.

## Figures and Tables

**Table 1:** Identification of 15 strains based on colony characterization and 16S rRNA sequence as well as assessment of protease and lipase production.

Bacterial Strains	Colony Characterization				16S rRNA Sequences			Enzyme Production	
	Morphology	Color	Texture	Margin	Blast	Similarity (%)	Accession No.	Protease	Lipase
SI-1	round	red	smooth, viscid	entire	*Serratia marcescens*	99.86	PX473135	+	−
SI-2	round	dirty white	smooth, viscid	entire	*Pseudomonas koreensis*	99.79	PX473136	−	+
SI-3	round	white	smooth, viscid	entire	*Serratia marcescens*	99.73	PX473137	+	−
SI-4	round	dirty white	smooth, viscid	entire	*Bacillus albus*	99.93	PX473138	+	−
SI-5	irregular	dirty white	rough, powdery	serrate	*Tsukamurella ocularis*	99.93	PX473139	+	+
SI-6	round	white	smooth, viscid	entire	*Serratia marcescens*	99.73	PX473140	+	+
SI-7	round	pale yellow	smooth, viscid	entire	*Stenotrophomonas maltophilia*	99.66	PX473141	+	−
SI-8	round	dirty white	smooth, viscid	entire	*Pseudomonas piscis*	99.93	PX474887	−	−
SI-9	round	dirty white	smooth, viscid	entire	*Pseudomonas allii*	99.50	PX474888	+	+
SI-10	round	pale yellow	smooth, viscid	entire	*Advenella kashmirensis*	99.64	PX473142	−	−
SI-11	round	dirty white	smooth, viscid	entire	*Pseudomonas piscis*	99.93	PX473143	+	−
SI-14	round	pale yellow	smooth, viscid	entire	*Advenella kashmirensis*	99.57	PX473146	−	−
SI-17	round	yellow	smooth, viscid	entire	*Stenotrophomonas maltophilia*	99.66	PX473149	+	−
SI-18	round	white	smooth, viscid	entire	*Serratia marcescens*	99.80	PX473150	+	−
SI-19	round	dirty white	smooth, viscid	entire	*Pseudomonas allii*	99.79	PX473151	+	−

(+): positive for enzyme production; (−): negative for enzyme production.

**Table 2:** The susceptibility of 15 strains to 10 different antibiotics.

Strains	KAN (mm)	GM (mm)	S (mm)	PEN (mm)	FLE (mm)	ROX (mm)	TET (mm)	FFC (mm)	IPM (mm)	RA (mm)
	30 μg/Piece	120 μg/Piece	10 μg/Piece	10 μg/Piece	5 μg/Piece	15 μg/Piece	30 μg/Piece	30 μg/Piece	10 μg/Piece	5 μg/Piece
SI-1	21.92 ± 0.38 c	24.83 ± 0.29 de	12.50 ± 0.25 e	6.00 ± 0.00 d	32.33 ± 0.29 a	6.00 ± 0.00 e	8.33 ± 0.14 h	12.25 ± 0.43 d	28.67 ± 0.29 e	10.67 ± 0.14 e
SI-2	23.64 ± 0.63 b	26.68 ± 0.25 c	12.23 ± 0.85 e	6.00 ± 0.00 d	23.02 ± 0.41 ef	6.00 ± 0.00 e	16.83 ± 1.02 de	6.00 ± 0.00 g	22.28 ± 0.37 g	11.95 ± 0.61 de
SI-3	20.72 ± 0.32 c	23.90 ± 0.23 e	17.59 ± 1.40 bc	6.00 ± 0.00 d	27.55 ± 0.08 cd	6.00 ± 0.00 e	6.72 ± 0.25 hi	11.95 ± 0.51 d	26.80 ± 0.44 f	8.68 ± 0.41 f
SI-4	19.46 ± 0.02 c	22.04 ± 0.57 f	17.86 ± 0.39 bc	13.52 ± 0.49 c	24.44 ± 0.21 e	22.98 ± 0.20 a	21.17 ± 0.20 c	28.06 ± 0.35 a	35.99 ± 0.20 c	16.72 ± 0.43 bc
SI-5	14.14 ± 0.67 d	23.88 ± 0.24 e	6.00 ± 0.00 f	11.26 ± 0.33 c	23.91 ± 0.23 ef	17.49 ± 1.03 b	10.68 ± 0.69 g	29.10 ± 0.95 a	37.83 ± 1.31 b	20.30 ± 0.59 a
SI-6	20.71 ± 0.16 c	24.63 ± 0.45 e	14.28 ± 1.26 de	6.00 ± 0.00 d	29.25 ± 0.52 b	6.00 ± 0.00 e	8.45 ± 0.34 h	12.61 ± 0.47 d	26.61 ± 0.19 f	8.91 ± 0.75 f
SI-7	6.00 ± 0.00 e	13.80 ± 0.23 g	6.00 ± 0.00 f	6.00 ± 0.00 d	17.59 ± 2.96 g	6.00 ± 0.00 e	16.68 ± 0.48 e	17.99 ± 0.25 c	6.00 ± 0.00 k	6.00 ± 0.00 g
SI-8	19.57 ± 0.48 c	22.07 ± 0.22 f	6.00 ± 0.00 f	6.00 ± 0.00 d	23.65 ± 0.28 ef	6.00 ± 0.00 e	17.06 ± 0.53 de	6.00 ± 0.00 g	15.13 ± 0.48 i	15.12 ± 0.45 c
SI-9	24.32 ± 0.07 b	26.55 ± 0.27 cd	14.30 ± 0.91 de	6.00 ± 0.00 d	24.44 ± 0.76 e	6.00 ± 0.00 e	13.76 ± 0.80 f	6.00 ± 0.00 g	13.66 ± 0.43 i	11.91 ± 0.39 de
SI-10	24.24 ± 0.65 b	28.63 ± 0.01 b	12.21 ± 0.84 e	18.67 ± 2.68 b	26.83 ± 1.30 d	7.88 ± 0.50 d	29.03 ± 0.44 b	24.69 ± 0.56 b	36.03 ± 0.63 c	17.23 ± 0.84 b
SI-11	20.64 ± 0.76 c	23.96 ± 0.69 e	6.00 ± 0.00 f	6.00 ± 0.00 d	22.52 ± 0.20 f	6.00 ± 0.00 e	10.99 ± 0.46 g	9.13 ± 0.62 f	11.99 ± 0.09 j	15.22 ± 0.62 c
SI-14	28.09 ± 1.42 a	37.85 ± 1.68 a	18.28 ± 1.02 ab	25.01 ± 0.11 a	29.54 ± 0.30 b	10.46 ± 1.65 c	32.28 ± 0.89 a	28.34 ± 0.13 a	44.36 ± 0.92 a	16.56 ± 0.22 bc
SI-17	6.00 ± 0.00 e	10.91 ± 0.29 h	6.00 ± 0.00 f	6.00 ± 0.00 d	23.29 ± 0.42 ef	6.00 ± 0.00 e	13.65 ± 0.59 f	19.45 ± 0.07 c	6.00 ± 0.00 k	13.34 ± 0.66 d
SI-18	23.48 ± 0.21 b	29.10 ± 0.53 b	20.22 ± 0.81 a	6.00 ± 0.00 d	31.80 ± 0.32 a	6.00 ± 0.00 e	6.00 ± 0.00 i	10.19 ± 0.77 ef	31.83 ± 0.15 d	7.00 ± 0.39 g
SI-19	26.96 ± 0.38 a	29.32 ± 0.13 b	15.91 ± 0.31 cd	6.00 ± 0.00 d	28.51 ± 0.30 bc	6.00 ± 0.00 e	18.62 ± 0.72 d	11.59 ± 0.57 de	18.02 ± 0.14 h	12.96 ± 0.40 d

Values that are separated by distinct lowercase letters within the same column indicate a significant difference at *p* < 0.05. KAN: kanamycin, GM: gentamicin; S: streptomycin; PEN: penicillin; FLE: fleroxacin; ROX: roxithromycin; TET: tetracycline; FFC: florfenicol; IPM: imipenem; RA: rifampin.

**Table 3:** Systematic comparison of microbial distribution between *Solenopsis invicta* and other ants in different geographical ranges.

Ant Species	Geographical Range	Dominant Microorganisms	Distribution	References
*Solenopsis invicta*	Zhejiang, China	*Stenotrophomonas*, *Serratia*, *Bradyrhizobium*, *Pseudomonas*, *Bradyrhizobium*, *Sphingomonas*, *Brucella*, *Luteibacter*, *Smaragdicoccus*	whole ant (sterilized)	This study
*Solenopsis invicta*	TX, USA	*Enterococcus*, *Enterobacter*, *Kluyvera*, *Lactococcus*, *Pseudomonas*, *Achromobacter*, *Bacillus*, *Serratia*, *Listeria*	midgut	[128]
*Solenopsis invicta*	LA, USA	*Klebsiella*, *Enterobacter*, *Pseudomonas*, *Acinetobacter*, *Serratia*, *Burkholderia*, *Pantoea*, *Providencia*, *Citrobacter*, *Morganella*, *Obesumbacterium*, *Lactococcus*	gut	[130]
*Solenopsis invicta*	Guangzhou, China	*Pseudomonas*, *Exiguobacterium*, *Acinetobacter*, *Mesoplasma*, *Bacillus*, *Proteus*	venom glands	[131]
*Solenopsis invicta*	Guangxi, China	*Mesoplasma*, *Exiguobacterium*, *Pseudomonas*, *Acinetobacter*, *Bacillus*, *Enterococcus*	venom glands	[131]
*Solenopsis invicta*	Guangzhou, China	*Enterococcus*, *Serratia*, *Lactococcus*, *Clostridioides*, *Bacillus*, *Pseudomonas*, *Brevibacterium*, *Enhydrobacter*	whole ant (sterilized)	[134]
*Solenopsis invicta*	Foshan, China	*Enterococcus*, *Weissella*, *Lactococcus*, *Brevibacterium*, *Bacillus*,	whole ant (sterilized)	[134]
*Solenopsis invicta*	Guangzhou, China	*Enterococcus*, *Lactococcus*, *Lactobacillus*, *Pseudomonas*, *Brevibacterium*, *Weissella*	whole ant (sterilized)	[134]
*Solenopsis invicta*	TX, USA	*Bacillus*	hemolymph	[129]
*Solenopsis invicta*	TX, USA	*Acinetobacter*, *Aeromicrobium*, *Conexibacter*, *Bacillus*, *Caldilinea*, *Bradyrhizobium*, *Marmoricola*, *Nocardioides*, *Stenotrophomonas*, *Mycobacterium*, *Pseudomonas*, *Streptomyces*	whole ant (sterilized)	[132]
*Solenopsis geminata*	TX, USA	*Acinetobacter*, *Bacillus*, *Pseudomonas*, *Propionibacterium*, *Conexibacter*, *Marmoricola*, *Nocardioidaceae*, *Nocardioides*, *Sphingomonas*, *Stenotrophomonas*	whole ant (sterilized)	[132]
*Pheidole nodus*	Zhejiang, China	*Holdemania*, *Wolbachia*	whole ant (sterilized)	This study

## Data Availability

The datasets presented in this study can be found in online repositories. The names of the repository and accession number can be found at: National Center for Biotechnology Information, PRJNA1414222.

## Supplementary Materials

The following supporting information can be downloaded at: https://www.mdpi.com/article/10.3390/microorganisms14040808/s1, Table S1: Ant samples sequencing and NCBI species identification.
