# Supplementary material for: Symbiotic Bacterial Diversity, Functional Profiling and Antibiotic Susceptibility of the Red Imported Fire Ant
Source: Microorganisms. 2026 Apr 1;14(4):808. doi: 10.3390/microorganisms14040808 (PMC13118498; doi:10.3390/microorganisms14040808)
Supplement: Supplementary file 1 [file microorganisms-14-00808-s001.zip › microorganisms-4212152-supplementary.pdf]

Table S1. Ant samples sequencing and NCBI species identification

Sample	Blast sequences	Identity (%)	E-value	Accession number
Group 1	*Solenopsis invicta*	100.00%	0.00	PX474348
Group 2	*Pheidole nodus*	99.40%	0.00	PX474349
